# Fabrication of Cu/SiC Surface Composite via Thermo-Mechanical Process (Friction Stir Processing) for Heat Sink Application

**DOI:** 10.3390/ma18051179

**Published:** 2025-03-06

**Authors:** Harikishor Kumar, Abhishek Agarwal, Michel Kalenga Wa Kalenga, Rabindra Prasad, Parshant Kumar, L. Aslesha Chilakamarri, Balram Yelamasetti

**Affiliations:** 1 Department of Mechanical Engineering, MLR Institute of Technology, Hyderabad 500043, Telangana, India; 2 Department of Mechanical Engineering, College of Science and Technology, Royal University of Bhutan, Phuentsholing 21101, Bhutan; 3 Department of Metallurgy, School of Mining, Metallurgy and Chemical Engineering, Faculty of Engineering and the Build Environment, University of Johannesburg, P.O. Box 17001, Doornfontein 2028, South Africa; 4 Department of Mechanical Engineering, Indian Institute of Information Technology, Design and Manufacturing (IIITDM), Jabalpur 482005, Madhya Pradesh, India; 5 School of Mechanical Engineering, Dr. Vishwanath Karad MIT World Peace University, Pune 411038, Maharashtra, India; 6 Department of Mechanical Engineering, Vignan’s Institute of Engineering for Women, Visakhapatnam 530049, Andhra Pradesh, India

**Keywords:** friction stir processing, SiC, hardness, tensile strength, wear, thermal conductivity

## Abstract

For the busting of heat, generated in electronic packages, relevant materials need to be developed. Metal matrix composites may be considered as an option to tailor the properties of a material (Cu) by incorporating an additional phase (SiC) for fulfilling the requirements of thermal management systems. The composite (Cu/SiC) was manufactured by friction stir processing. For good interfacial strength, the biggest challenge in the fabrication of Cu/SiC composite was to abolish the reaction between Cu and SiC. Being solid in nature, the process (friction stir processing) does not allow temperature to reach the interfacial interaction. Scanning electron microscopy, electron backscattered diffraction, and optical microscopy were used to characterise the composite for microstructural features (particle dispersion, phases present). To confirm the presence of reinforcement, EDS analysis was also performed on the composite. Results indicated the presence of Cu and SiC phases in the stir zone (SZ) with uniform and homogeneous separation of reinforcements. The composite displayed higher hardness, tensile strength, and wear resistance in comparison to unprocessed copper. However, ductility decreased due to high hardness in the composite.

## 1. Introduction

Friction stir processing (FSP) does not involve melting of materials during processing irrespective of the chosen process parameters. Although it has been substantiated from friction stir welding (FSW) developed by The Welding Institute (TWI) in 1991 [1] and works on the same principle but has separate existence as a thermomechanical processing technique. The process is well accepted in the scientific community for microstructural refinement, densification, and homogeneity. The process consists of a tool generally of non-consumable nature, which rotates on its axis with high rotational speed. As the tool shoulder remains in contact with the material, it leads to the generation of heat and healthy stirring and mixing. Due to strenuous stirring and mixing of the materials during processing, it has interesting applications in the improvement of microstructural features and homogenisation of cast alloys and powder metallurgy-fabricated components [2,3,4,5,6,7] and production and homogenisation of metal matrix composites [8,9,10,11,12,13,14].

Harpreet Singh Arora et al. [2] processed Mg-based alloy with multiple FSP passes and observed drastic change in microstructural features of the composite. They also reported almost two-fold increase in the hardness. Sub-grain boundary pinning due to nanoparticles were cited as the main strengthening mechanism. In another study on Mg alloy (AM60B), Cavaliere et al. [3] studied the effect of FSP on mechanical properties and microstructure features. Tensile test was conducted at room temperature as well as at higher temperature (150–300 °C) under different strain rate conditions. The study showed substantial grain refinement and hence an appreciable improvement in strength of the composite with respect to its counterpart unprocessed alloy. Christian B. Fuller et al. [4] friction stir processed a 5083-H321/5356 ARC-welded structure and analysed the effect on microstructure and mechanical properties. The result showed an improvement in not only tensile strength but also in percentage elongation. M. D. Fuller et al. [5] studied the effect of fusion welding and friction stir processing on microstructural and mechanical transformation of cast NiAl bronze. Keiichiro Oh-Ishi et al. [6] processed the alloy NiAl bronze with FSP and studied the microstructure features in detail. Masato Tsujikawa et al. [7] applied FSP on Mg—5.5 mass %Y—4.3 mass %Zn Cast Alloy for high strengthening. Y. Morisada et al. [13] reinforced MWCNTs in AZ31 for the fabrication of surface composite by FSP and recorded the hardness. The hardness of the composite was almost double of the AZ31 alloy attributed to grain refinement. C. J. Lee et al. [14] incorporated SiC (5–10 vol%) in Mg alloy in order to develop a surface composite.

Due to the solid-state nature of the process, it almost eradicates all the defects associated with fusion-based techniques and hence is popular for composite fabrication. Moreover, unlike the other conventional techniques, it is capable of controlling the depth of processing through penetration of the tool pin [15]. For composite fabrication, particles (reinforcements) are pre-placed on the plates by any of the exiting strategies (groove making, blind holes, etc.), and once the particles are properly settled inside the holes or grooves, it is closed with the tool (no pin) to stop their sputtering during processing and finally processed for composite fabrication.

Recently, materials with high electrical and thermal conductivities and capability to maintain the strength at high temperature are in demand for thermal management systems [16]. Copper and its alloys possess the required properties but have some inherent drawbacks like low hardness, wear resistance, tensile strength, and fatigue [17,18,19]. Hence, tailoring the properties of copper and their alloys for a wide range of applications is of prime importance. Primarily, age hardening and insertion of hard particles in the matrix (Cu) are in trend. The issue with the age-hardenable copper alloys is precipitate coarsening at high temperature and hence loss of strength. In this regard, although the presence of reinforcements in in copper matrix reduces density, ductility, and electrical and thermal conductivities but does not suffer precipitate coarsening at high temperature. Moreover, the reinforcements such as oxides, carbides, nitrides, etc. used in Cu are insoluble and thermally stable at high temperatures.

Copper–silicon carbide has been the centre of attraction globally because it possesses high thermal and electrical conductivities along with good mechanical and wear properties [20,21,22,23]. Therefore, they are not only a strong candidate as a component in thermal packaging but also as an electrical contact in relays, switches, circuit breakers, railway overhead current collectors, etc. [24,25,26]. Copper reinforced with particles (TiC, Al_2_O_2_, SiC, B4C, etc.) [27,28] are termed as copper matrix composites. Among various ceramic particles, SiC particles are considered fascinating due to its properties like high wear resistance, elastic modulus and excellent oxidation and corrosion resistance along with good mechanical properties [29]. Also, it is appropriate cost-wise for consideration as reinforcement.

Ceramic particles can be incorporated in the metal matrix through various solid- and liquid-based processes such as powder metallurgy [30], mechanical alloying [31], in situ reactions [32], spark plasma sintering [33], spray forming [34], etc. It is very difficult to control defects like porosity [35], non-uniform distribution [36], and clustering [27] and interfacial reactions during these processes. Nonetheless, FSP does not include such defects if properly handled [37,38,39] because of its solid-state nature.

Although the literature is available on the fabrication of copper-based surface composites by using SiC as reinforcement via FSP, but detailed analysis of the mechanism involved is yet to be discussed. Keeping the above in mind, the current study focuses on the incorporation of SiC in copper through friction stir processing and investigates the effect on microstructure, mechanical, and tribological behaviour along with detailed analysis of the fractography and wear mechanism involved.

## 2. Materials and Methods

**Figure 1:**
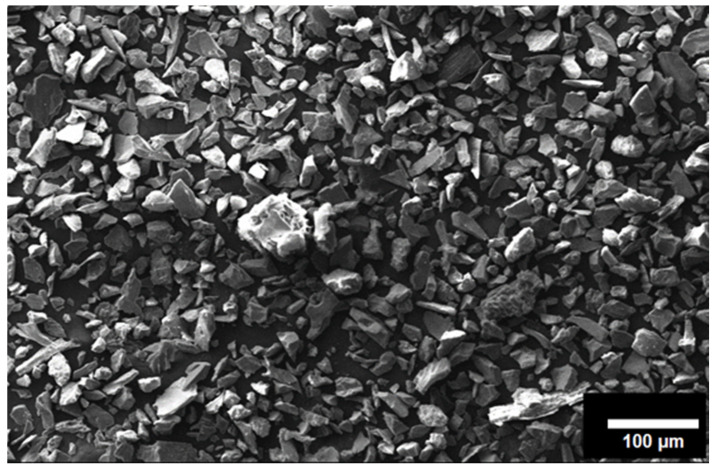
SEM micrograph of the reinforcement (SiC).

For the experimentation, SiC and commercially pure copper was used. The average size of the reinforcement (SiC) particle was estimated as 2–8 µm. The matrix copper was in the hot-rolled form. The SEM image of the SiC particle is given in Figure 1.

**Figure 2:**
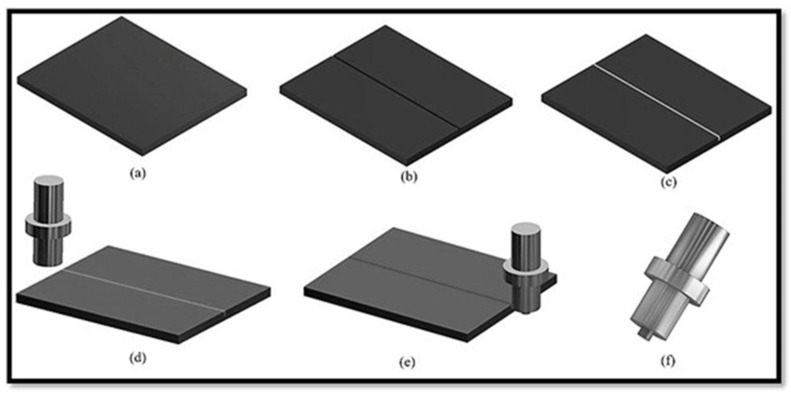
Schematic diagrams for composite fabrication via FSP. (**a**) The plate of known dimension (**b**) Groove making on the surface of the plate (**c**) Filling of particles in the groove (**d**) Closing of the groove with a pin-less tool (**e**) Processing of the plate (**f**) The tool with a pin used for processing.

Samples of dimension 200 mm × 100 mm × 6 mm were machined from a copper plate for experimental requirement. For pre-placement of the particles, a rectangular cross-section groove was machined in the centre of the plate along length using wire EDM. The depth of the groove was kept 3.5 mm, whereas for width, the theoretical volume fraction formula was employed. The width was estimated to be 1.5 mm, which was for 18 vol% of reinforcement. The groove was filled with particles, and to ensure that it is tightly filled, plates were shaken multiple times. The groove opening was closed with a tool (pin-less) so that sputtering of particles during processing and hence wastage can be avoided during processing. Finally, it was processed for composite fabrication. In the current experimentation, a modified vertical milling machine was used. An axial load of 10 kN, traverse speed of 35 mm/min, and 1150 RPM rotational speed were used. The tool was also tilted from the vertical by 2.5 degrees. The process parameters were decided on the basis of the literature and authors’ past experience [37,38,39,40,41]. Kumar et al. [37,38,39] used FSP as a processing technique to process copper-based surface composites. They reported that the traverse speed of 35 mm/min, 1150 RPM rotational speed, and 2.5 degrees tilt angle are the optimum process parameters for the processing of copper surface composites by FSP. The tool used for processing was super alloy IN718 in the peak-aged form [42]. The tool pin diameter was kept at 6 mm and its length at 4 mm, keeping the groove depth in mind. The tool shoulder was flat with a diameter of 18 mm. The environmental conditions were kept normal during the processing of the composite. The composite fabrication process from start to end is schematised in Figure 2.

The specimens were machined according to different ASTM standards for the characterisation. For microstructure, machined specimens were moulded to hold it properly during disc and cloth polishing. Specimens were polished following ASTM standard E3-11 [43] before etching with 40 g of chromic acid, 4 g of sodium sulphate, 3.4 mL HCL in 200 mL distilled water solution. A Mitutoyo micro-hardness tester was used for hardness estimation of the samples. Processed plates were machined from top by 1 mm and then were polished properly before indentation. The indentation was captured along transverse section of the processed plate. Fair distance between the indentations was maintained. At every place three indentations were averaged to plot the hardness profile. The load and dwell time were kept at 100 g and 15 s, respectively. Hardness measurement was performed according to the ASTM E-384 standard [44]. Microstructural features were captured using an optical microscope (Serial no.-DEW/101, DEWINTER Technologies, Milan, Italy), a scanning electron microscope (Serial no.-EVO18-20-45, ZEISS EVO 18 RESEARCH, Oberkochen, Germany), and an electron backscattered diffraction technique (EBSD). The SEM equipped with EDS was used for compositional analysis of the composites. The worn-out surfaces of the wear-tested specimens were also observed under the SEM for the significant wear mechanism involved. Hardness of the fabricated composites and base copper was observed using a Vickers microhardness tester (Model-SEMIAUTOMATIC, Serial no.-104, MICROMACH Technologies, Pune, India). For hardness measurement, specimens were extracted from the processed plate across the processing direction. Before indentation, specimens were polished with different emery papers. A load of 300 gm was used for an indentation period of 15 s. The strength (tensile) was tested by an Instron 5982 (Grove City, PA, USA). The sample prepared was in a dog bone shape according to ASTM (ASTM: E8/E8M-16a [45]). All the samples were tested at room temperature.

The wear test was conducted in a dry sliding condition. The environmental condition was atmospheric with an average temperature of 33 ± 2 °C. The apparatus was supplied by DUCAM-Bangalore. The samples for the test were extracted from base copper and fabricated composites. The samples were in the form of tablets. To hold it during the test, a specially designed holder was fabricated. The samples were slid against EN31 steel, which had a hardness of approximately 65 HRC. Emery papers of different grit size were used to polish the samples. Care was also being taken to remove the oil or grease (if any) from the sample and disc with acetone. During the test, specimens were slid against the disc for 1000 m at a constant radial speed of 1.5 m/s. The load was also kept constant during the test at 30 N. Cumulative weight loss suffered by the specimens during the test was plotted against distance for wear-loss calculation. Additionally, a hardness tensile test was also conducted to find out the strength of the composite. The uni-axial tensile tests were performed at room temperature at a strain rate of 1 × 10^−3^ s^−1^. The tensile tested specimens were observed under the SEM for fractography.

## 3. Results and Discussion

### 3.1. Macrostructure and Microstructure

**Figure 3:**
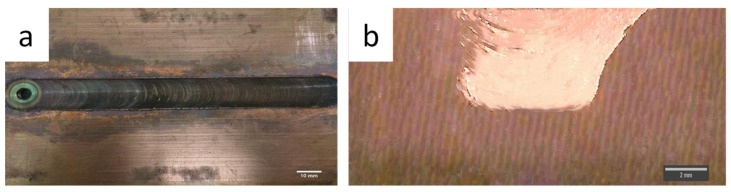
(**a**) top view of the processed plate (**b**) cross-sectional macrostructure.

The top macroscopic view of the processed plate captured through the optical microscope is depicted in Figure 3.

A ring-like appearance without depression or discontinuity can be observed on the surface. The ring-like feature on the surface of the processed plate is the characteristic feature of FSP and reflects the sound processing and is essential for defect-free composite fabrication. The formation of ring-like texture on the surface of the plate is because of simultaneous motion (linear and rotational) of the tool. During FSP, materials experience severe plastic deformation and stirring from the advancing side of the tool pin to the retreating side of the tool pin, which finally results in the form of a ring. Moreover, a smooth surface without defects reflects the appropriate selection of process parameters during the process, which could sufficiently plasticise the materials by its easy movement from the advancing side to the retreating side.

Figure 3b is for the cross-sectional macrostructure of the composite. As mentioned, particles that were initially pre-placed in the groove vanished completely. Furthermore, the macrostructure defects such as worm holes or tunnels are also not visible. This, we can assume, is as a consequence of the appropriate selection of process parameters. Material flow during the process depends on the plastic state of the materials. In the present case, there is no visible defect in the macrostructure and hence indicates the sufficient plasticisation and stirring of the material during processing, which could promote the smooth flow of the material from the advancing to retreating side and competition of composite fabrication. The shape of the stir zone is basin like (Figure 3b). The shape of stir zone is largely affected by the tool shoulder and pin. The tool shoulder had a larger diameter (18 mm), which affected the material largely at the top, whereas the pin, being smaller in diameter (6 mm), could affect only little and hence the shape is like a basin.

**Figure 4:**
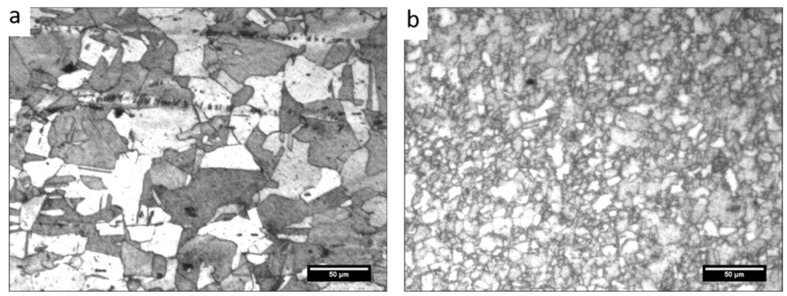
Microstructure of (**a**) base copper and (**b**) the processed plate without reinforcement.

Microstructural characterisation of base copper and processed copper without reinforcement captured by the optical microscope is presented in Figure 4.

As can be observed from the micrograph (Figure 4a), the grain is coarse and elongated at some places. Moreover, the microstructure also comprises strain twins, which appear due to high-stacking fault energy possessed by copper. The average size of the grains is larger. However, in the processed plate, the grain is fine and equiaxed. Also, the size of the grain has reduced drastically. The rolled copper plates used for experimentation had an average grain size of 34 µm, and after processing, it reduced to 2.7 µm. During FSP, mechanical deformation and a temperature rise due to friction are bound to happen simultaneously. This synergetic effect of deformation and heat generation causes dynamic recrystallisation and hence grain refinement.

**Figure 5:**
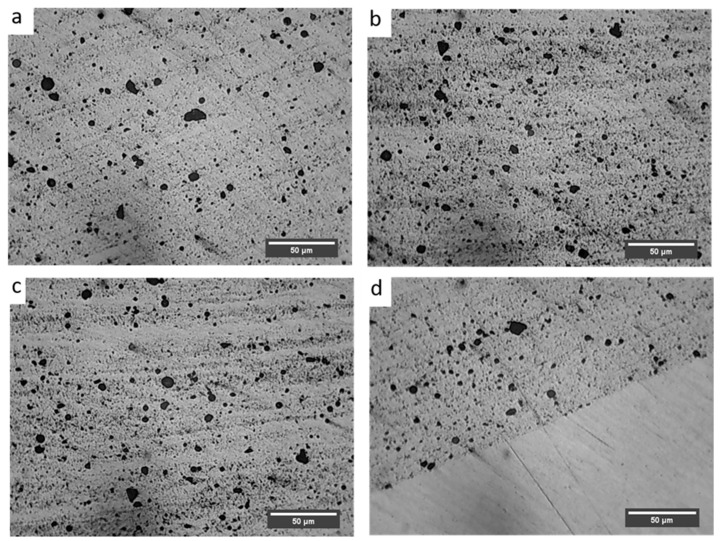
Optical images of the nugget zone captured (**a**) at the top of the zone, (**b**) at the bottom of the zone, (**c**) at the centre of the zone, and (**d**) at the interface between reinforced and non-reinforced.

Figure 5 depicts microstructural features of the developed Cu/SiC surface composite via optical microscopy.

**Figure 6:**
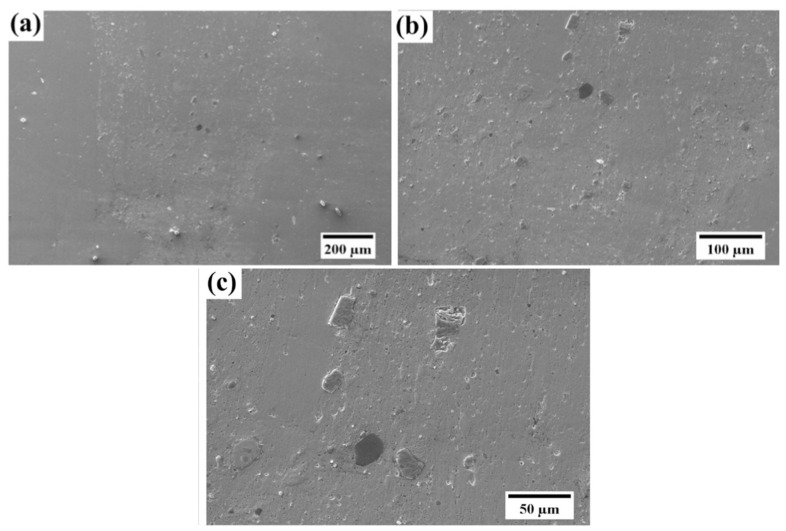
SEM images of the nugget zone of the composite at different magnifications (**a**) 500×, (**b**) 1000× and (**c**) 2000×.

It is evident from the microstructure, the SiC particles are distributed throughout the copper metal matrix. The image was captured at the various sections of the processed plate to justify the fact that particles are everywhere. Moreover, the particles are almost placed equidistant from each other, i.e., the particle free space is not visible in the micrograph. In processes where solidification involves, the scenario is generally reversed, i.e., particle segregation along the grain boundaries is hard to control [46]. Segregation of particles in phenomena like stir casting is because of the difference in densities of the reinforcements and the matrix. In the present study, nothing like that was observed, which is solely because the process is having solid nature where solidification did not occur. The process (FSP) involves severe plastic deformation of the matrix without melting. As the matrix is not in the molten phase, reinforcements are not free to move freely either at the top or the bottom due to density difference. Hence, the developed composite is free from the segregation of Si particles. Additionally, the particles are dispersed homogeneously in the copper matrix. Also, the complete stir zone is engulfed in the particles. Agglomeration of particles or particle free space is not visible in the microstructure. The processing parameters such as blade speed, tool rotational speed, tilt angle, axial load, etc. play a vital role in the dispersion of particles in the matrix [47]. So, it can be safely assumed that the chosen parameters for the present experimentation were appropriate. Homogeneous sputtering of particles in the matrix is essential for better performance of the composite including isotropic properties. Microstructural features of the composites were also captured through the SEM (Figure 6).

Microstructure characteristics are also clear from the SEM micrograph. Here, it can also be seen that the particles are uniformly and homogeneously scattered throughout the matrix. There is no sign of segregation and clustering of particles in the matrix. Although, the grain boundaries are not even clearly visible in the SEM micrograph but due to the absence of segregation, the particle distribution may be assumed in transgranular. The possibility of some particles being on the grain boundaries cannot be denied. FSP comes under the category of severe plastic deformation processes. During the processing of the composites, the matrix experiences severe plastic deformation and hence a high-strain field develops in the materials. The matrix being elastic in nature is capable of absorbing the stress but reinforcements suffer fracture due to their brittleness. It also happened with SiC particles in the present investigation, which can be observed by comparing Figure 1 and Figure 5. Owing to the high-strain field developed and the stirring action of the tool, particles which had an irregular shape, get fractured during processing. After closely observing the SEM micrograph of SiC in Figure 1, it can be inferred that particles are not having a regular shape. During processing, as the material flows from the advancing side to the retreating side, the particles having an irregular shape will resist the motion more and hence stress development will be more prominent in those particles. Due to their brittle nature, they did not absorb the stress, and fracture ensued. Another reason for fracture may be cited as a high RPM of the tool during processing. The particles that came in contact with the tool get fractured. It is important to note that the fractured particles will have smaller size, which is difficult to reinforce uniformly in the matrix with a single pass of FSP. Still, particle agglomeration was not observed in the composite.

Further, for the assessment of the interface between the reinforcement and the copper matrix, the SEM image at high magnification was captured (Figure 6c). From the image, it can be inferred that the bonding between the matrix and the reinforcement is excellent because there are no irregularities at the interface. The interface is clean, and no voids are there, which further justifies the strong bonding between SiC and the copper material.

**Figure 7:**
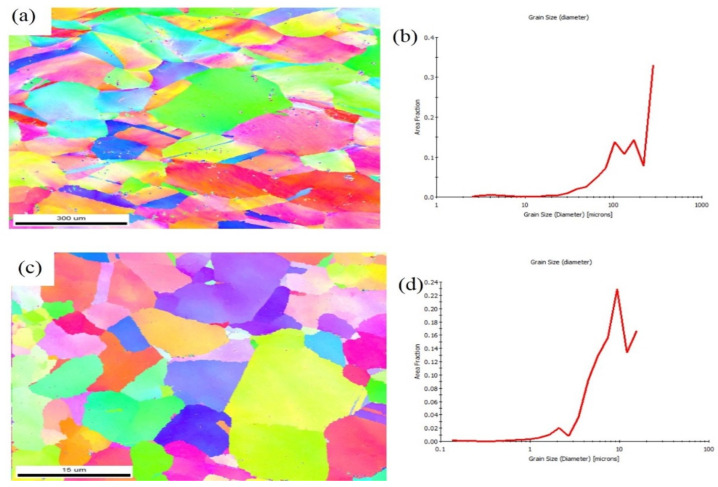
EBSD images of (**a**) base copper and (**c**) composite and grain size distribution (**b**,**d**).

The EBSD images of the copper matrix and the produced Cu/SiC composite are presented in Figure 7.

The micrograph shows that the copper matrix experienced drastic grain refinement during composite fabrication. Also, the grains are equiaxed. The estimated average grain size of the base copper was 34 µm, which reduced to 2.7 µm after fabrication. The following factors may be considered responsible for grain refinement: materials experiencing enormous deformation and a temperature rise during fabrication. Simultaneously, these factors lead to dynamic recrystallisation [47] and hence reduction in grain size. Another reason is the reinforcement (SiC), which brought about the Zener effect in the picture. The pinning effect of reinforcement created Zener drag, which restricted the growth of recrystallised grains. As mentioned earlier, particles that are uniformly and homogeneously scattered in the matrix provided more pinning effects.

### 3.2. Compositinal Analysis

**Figure 8:**
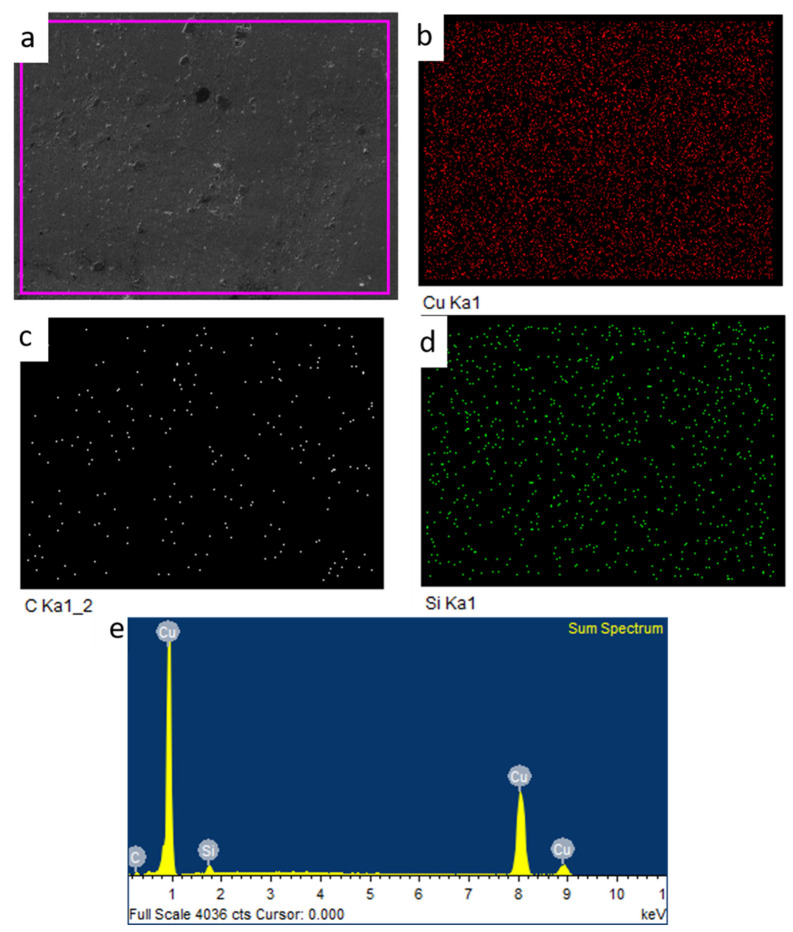
(**a**) The SEM image of the processed section of the composite (**b**–**e**) the EDS maps of the elements and their distribution.

The compositional mapping of the composite was performed through EDS mapping, which is depicted in Figure 8.

The EDS analysis conducted for the composite clearly shows that the SiC particles are there in the composite. Moreover, it is also obvious from the analysis that except for the matrix and reinforcement, nothing is there. Absence of picks of any discernible products from the analysis indicates that there was no initiation of interfacial reactions between the matrix and reinforcement. In FSP, the temperature does not reach the point of initiation of any reaction between the reinforced materials and the matrix. Moreover, the high strain rate possessed by the process and its short duration during processing also did not allow enough time for atomic diffusion.

## 4. Mechanical Properties

**Figure 9:**
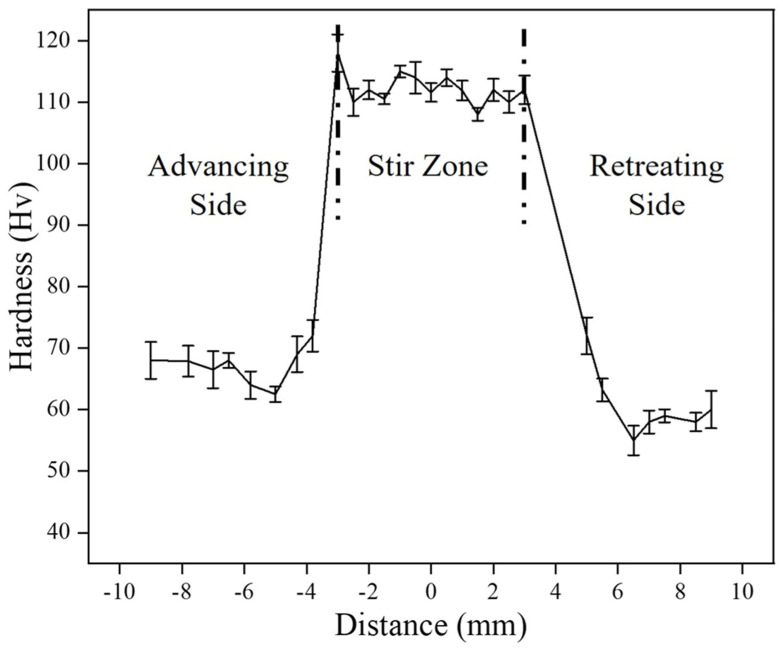
Microhardness profile of the composite.

Figure 9 is for the microhardness profile of the composite measured across the processed zone.

The average hardness value for the base copper was estimated to be 66 HV, whereas the fabricated composite was found to have exhibited higher hardness (114 HV). The hardness value has decreased slightly in the heat-affected zone. Annealing effect may be responsible for this decrement. In the heat-affected zone, dislocation density decreases, which might have further reduced hardness. As discussed earlier, FSP leads to grain refinement due dynamic recrystallisation. Because of small grains in the material, the number of grain boundaries will increase, which act as an obstacle for the free movement of dislocations. As dislocations are not free to move thus hardness increased. Other strengthening mechanisms like Orowan strengthening, the shear lag mechanism, and difference in the thermal coefficient mechanism [48,49] play an important role in the hardness improvement of the composite. The small, fractured particles of reinforcement brought about the pinning effect in the scene and stopped or delayed the movement of dislocation, which increased hardness. The interfacial integrity displayed an important role in transferring the load from soft copper to hard SiC, which bore the load more effectively and thus hardness increased. The additional dislocations developed owing to the difference in thermal coefficients either deflated or created a hindrance to dislocations, which also caused an improvement in hardness. A similar trend of hardness was observed by H. Kumar et al. [37].

**Figure 10:**
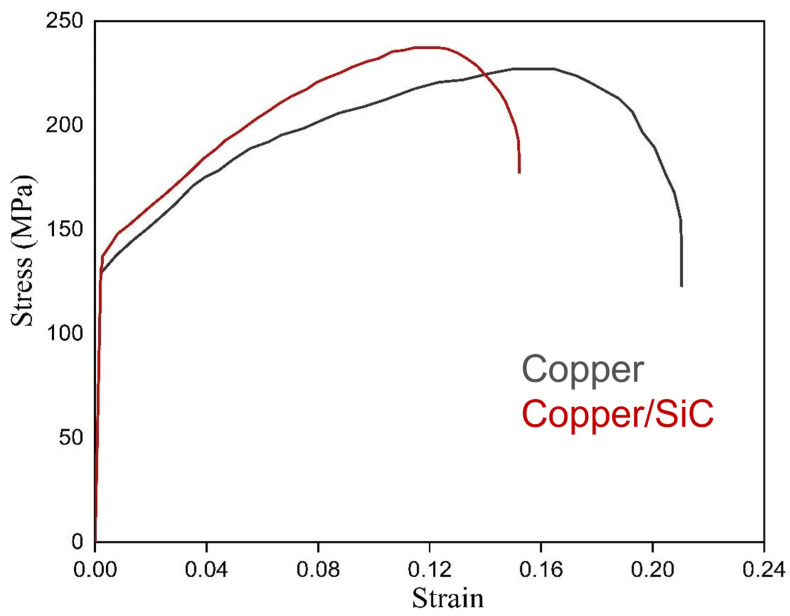
Stress–strain curve for the composite and base copper.

The effect of particle incorporation (SiC) in the copper matrix was further checked through tensile testing. The stress–strain curve for the composite and base copper is depicted Figure 10.

The result showed an almost 27% increment in ultimate tensile strength. The increment in yield strength was less and was estimated to be approximately 8%. The ductility measured as percentage elongation decreased for the composite with respect to base copper. The obtained date after the tensile test is given in Table 1.

The reason for the strength improvement of the composite may be cited as same as hardness like combination of microstructural refinement, excellent interfacial bonding, absence of defects, and work hardening. Besides the mechanism involved because of reinforcement, grain refinement and annealing effect also play their roles in strengthening the composites. Due to simultaneous activities of high deformation and a temperature rise, recrystallisation comes into the picture, which promotes grain refinement. But, at the same time, annealing effect is also there. So, whichever is the dominant factor affects the strength accordingly. The reduction in percentage elongation may be due to smaller grains in the composite, which could not provide sufficient space for dislocation movement during straining. The reinforcement may act as stress concentrators, which initiate cracks.

### Fractography

**Figure 11:**
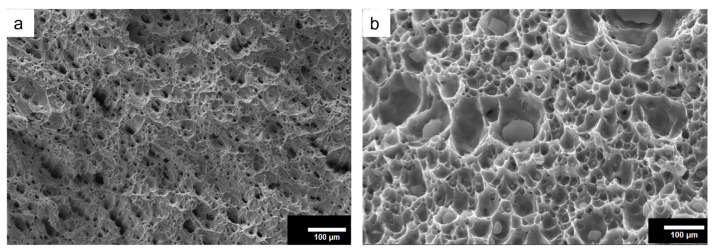
The SEM micrograph of fractured surfaces of (**a**) base copper and (**b**) the composite.

Figure 11 depicts the SEM images of fractured surfaces of tensile-tested specimens.

From the SEM micrograph (Figure 11a), dimples are visible, which is also obvious because copper is a ductile material. However, in the case of the composite, the opening is wide, which clearly indicates the reduction in percentage elongation. Moreover, the shiny and flat facets may also be observed from the fractured surfaces of the composite because of its slightly brittle nature. There is no sign of particles pulling out or cracks through particles, which indicates the strong bonding between particles and the matrix, also justified by the tensile result.

## 5. Wear Properties

**Figure 12:**
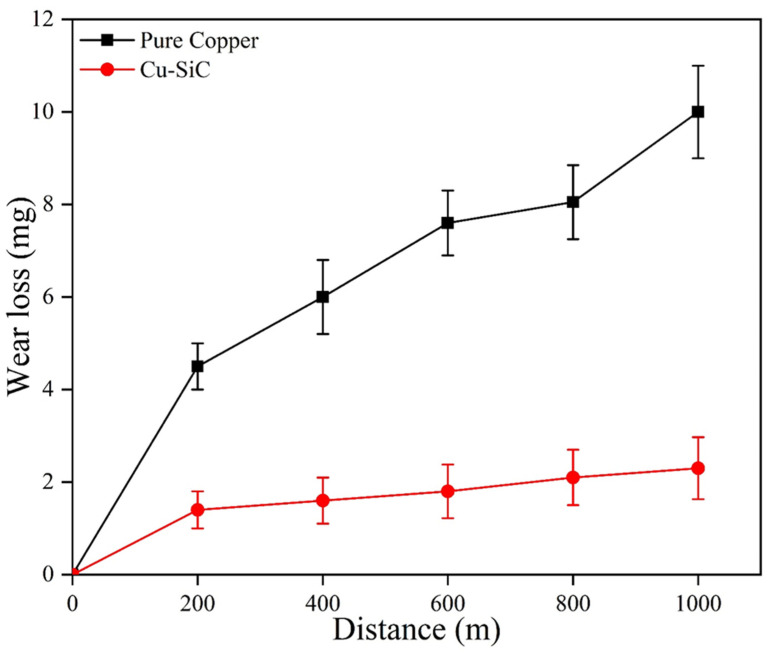
The wear rate profile of base copper and the composite.

Wear loss of the base copper and the developed composite with respect to the distance slid is provided in Figure 12 in a comparative manner.

**Figure 13:**
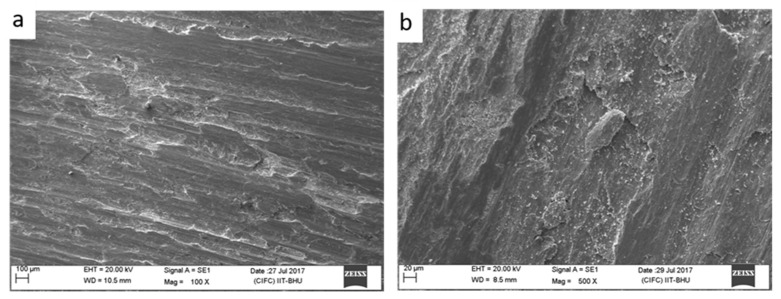
SEM morphology of worn-out surfaces of (**a**) base copper and (**b**) the composite.

It may be observed that with an increase in distance, the wear loss is increasing in both base copper and the composite. This gradual increase in wear loss with distance may be attributed to the melting of wear specimens. As samples slid on the counter, surface temperature will rise with respect to the increase in slid distance and hence the melting of the specimens. The wear loss of the composite has substantially reduced in comparison to base copper. This reduction in wear loss in the composite with respect to base copper may be attributed to the presence of hard SiC particles in the composite: their increased hardness. According to Archard’s law, hardness is directly proportional to wear resistance. Other mechanisms like grain refinement and work hardening may also be considered as a reason for higher wear resistance in the case of the composite. The hard SiC particles dispersed uniformly in the copper matrix may also be considered as one of the reasons behind the wear resistance improvement. These uniformly distributed particles will carry the load applied and also prevent the cutting of pins from the counter materials [50]. The worn surface morphology of base copper and the composite at speed 1.5 m/s and with a load of 30 N is depicted in Figure 13.

As can be observed, morphology for base copper and the composite is different. Hard asperities on the counter face (disc) easily penetrated through the soft surface of base copper and created grooves, and micro-pores can be observed from the figure. In contrast, the fabricated composite being hard did not allow asperities to penetrate through, and hence the grooves are narrow and thus also less material removal. It is justified by the flat surfaces of the composite. Delamination and deep craters are absent in the case of the composite because the hard surface of the composite did not allow the asperities to penetrate inside.

## 6. Conclusions

In this study, SiC particles were incorporated into the surface of copper materials by friction stir processing. The processed composite was studied comparatively for the microstructure and mechanical and tribological behaviour with respect to base copper. The outcomes are summarised below:

- The Cu/SiC composite was successfully fabricated using friction stir processing (FSP) without any defects.
- The SiC particles were uniformly and homogeneously distributed throughout the stir zone, with no signs of agglomeration or segregation along grain boundaries, which can be attributed to the solid-state nature of FSP.
- The stir zone hardness of the composite increased by 57% compared to base copper. However, a slight decrease in hardness was observed in the heat-affected zone (HAZ), likely due to annealing effects and a reduction in dislocation density.
- The ultimate tensile strength (UTS) of the composite improved by 13%, while elongation decreased by 35%, primarily due to grain refinement, the pinning effect, and the shear lag mechanism.
- The composite exhibited enhanced wear resistance, which can be attributed to its greater hardness, the presence of hard ceramic reinforcements, and their uniform dispersion within the matrix.

## Figures and Tables

**Table 1:** Numerical values for UTS, YS, and %EL after tensile testing.

Samples	UTS (MPa)	YS (MPa)	%EL
Base copper	211	131	23
Composite	243	141	15

## Data Availability

The original contributions presented in this study are included in the article. Further inquiries can be directed to the corresponding authors.
